# Temperature Driven Membrane Lipid Adaptation in Glacial Psychrophilic Bacteria

**DOI:** 10.3389/fmicb.2020.00824

**Published:** 2020-05-14

**Authors:** Noor Hassan, Alexandre M. Anesio, Muhammad Rafiq, Jens Holtvoeth, Ian Bull, Abdul Haleem, Aamer Ali Shah, Fariha Hasan

**Affiliations:** ^1^Applied Environmental and Geomicrobiology Laboratory, Department of Microbiology, Quaid-i-Azam University, Islamabad, Pakistan; ^2^Bristol Glaciology Centre, School of Geographical Sciences, Faculty of Science, University of Bristol, Bristol, United Kingdom; ^3^Department of Environmental Science, Aarhus University, Roskilde, Denmark; ^4^Department of Microbiology, Balochistan University of Information Technology, Engineering and Management Sciences, Quetta, Pakistan; ^5^Organic Geochemistry Unit, School of Chemistry, Cantock’s Close, University of Bristol, Bristol, United Kingdom

**Keywords:** cell membrane, cold adaptation, fatty acids, non-polar glaciers, psychrophilic bacteria

## Abstract

Bacteria inhabiting non-polar glaciers are exposed to large variations in temperature, which significantly affects the fluidity of bacterial cell membranes. In order to maintain normal functions of the cell membranes, psychrophilic bacteria adapt by changing the composition of cell membrane fatty acids. However, information on the exact pattern of cell membrane adaptability in non-polar low-temperature habitats is scarce. In the present study, 42 bacterial strains were isolated from the Ghulmet, Ghulkin, and Hopar glaciers of the Hunza Valley in the Karakoram Mountain Range, Pakistan and their cell membrane fatty acid distributions studied, using gas chromatography/mass spectrometry (GC-MS) for the analysis of fatty acid methyl esters (FAMEs) liberated by acid-catalyzed methanolysis. Furthermore, Gram-negative and Gram-positive groups were grown under different temperature settings (5, 15, 25, and 35°C) in order to determine the effect of temperature on cell membrane (CM) fatty acid distribution. The analyses identified the major groups of cell membrane fatty acids (FA) as straight-chain monounsaturated fatty acids (*n*-MUFAs) and branched fatty acids (*br*-FAs), accounting for more than 70% of the fatty acids analyzed. The distribution of br-FAs and *n*-FAs in bacterial cell membranes was significantly affected by temperature, with the level of br-FAs decreasing relative to *n*-FAs with increasing temperature. Notably, the production of polyunsaturated fatty acids (PUFAs) was only seen at lower temperatures. This study contributes to understanding, for the first time, the role of br-FAs in the maintenance of cell membrane fluidity of bacteria inhabiting non-polar habitats.

## Introduction

By far, the largest part of the biosphere on Earth, which includes the oceans, is exposed to temperatures below 5°C either perennially or seasonally (Margesin et al., 2007; Margesin and Miteva, 2011; Hoshino and Matsumoto, 2012). Arctic and Antarctic habitats, ranging from high mountains to the deep ocean, experience subzero temperatures as they are deprived of direct sunlight. Among these cold habitats the deep sea represents the major component (almost 71% of the Earth is covered by ocean), with 90% of the volume of the ocean having a temperature of ∼5°C. Other large cold habitats include snow, permafrost, sea ice and glacial habitats (35, 24, 13, and 10% coverage of the Earth surface, respectively) in addition to various other cold habitats such as lakes, cold soils (especially surface soils), cold deserts, and caves (Singh et al., 2006; Margesin and Miteva, 2011).

Bacteria inhabiting cold environments face prolonged frigid temperatures and daily freeze-thaw cycles (Montiel, 2000). They have to cope with special challenges to thrive under subzero temperatures, such as slow chemical reaction rates and limited enzyme activity, denaturation of proteins, increased water viscosity, decreased cell membrane fluidity (Russell, 1990; Crowe et al., 1992; Hassan et al., 2016) and limited water availability as a solvent for biochemical reactions (Wynn-Williams, 1990). However, bacteria have remarkable adaptive abilities that enable them to survive harsh and highly variable environmental conditions, including changes in pH and temperature (Ganzert et al., 2011; Bajerski and Wagner, 2013). These adaptation mechanisms include the expression of heat/cold shock proteins, production of protective compatible solutes or an altered metabolism (Georlette et al., 2004). Another strategy employed by bacteria is the modification of the cell membrane structure as it is involved in important metabolic processes and is a vital interface in the electron transport chain (Denich et al., 2003).

Homeoviscous adaptation, i.e., the alteration of cell membrane composition to maintain cell membrane fluidity in response to environmental changes, has long been observed in bacterial communities (Sinensky, 1974) and in bacteria involved in the direct synthesis of fatty acids, with diverse melting points, to maintain the required physical properties needed of membrane lipids (Mansilla et al., 2004). An increase in the amount of low-melting-point fatty acids (such as monounsaturated fatty acids, polyunsaturated fatty acids and branched-chain fatty acids relative to their saturated straight analogs) provides adequate membrane fluidity in almost all organisms (Fulco, 1983; Hazel and Williams, 1990; Suutari and Laakso, 1994). Low temperature and high pressure play a significant role in probing the integrity of membranes, with evidence even for irreversible change, i.e., from a fluid to a rigid state (Cossins and MacDonald, 1984; Hazel and Williams, 1990). Bacterial adaptation to low temperature and high pressure typically involves an increased proportion of branched chain fatty acids (BCFA) and unsaturated fatty acids (UFA) into membrane phospholipids (DeLong and Yayanos, 1986; Wirsen et al., 1986; Nichols et al., 1997). However, to the best of our knowledge, these results have only been obtained by studying Gram-negative bacteria residing in deep sea or a very few Antarctic habitats. The current study aims to characterize the cell membrane fatty acid profiles of Gram-positive and Gram-negative psychrotolerant bacteria isolated from non-polar glaciers and determine the role of saturated and unsaturated, straight and BCFAs in bacterial adaptation to low temperatures.

## Materials and Methods

### Sampling, Isolation and Identification of Bacterial Strains

Samples (ice, melted water, and sediment) were collected from three different glaciers, Ghulkin (36.42791 N, 74.80659 E), Ghulmet (36°12.474 N, 74°29.035 E) and Hopar (36.2108228 N, 74.7724664 E) of the Hunza Valley, Karakoram Mountains Range, Pakistan using Nasco Whirl–Pak bags and bottles (Fisher Scientific). A total of 42 different bacterial strains were isolated using R2A and Nutrient agar at 5, 15, and 35°C as incubation temperatures. Culture of each bacteria isolate was then preserved in 35% glycerol and stored at −20°C prior to further analysis.

The genomic DNA of all the bacteria isolates was extracted using the Invitrogen PureLink Microbiome DNA Kit (Invitrogen) following instructions given by manufacturer. After that 16S rRNA gene for all of the bacterial isolates was amplified using primers 27F and 1492R. PCR conditions used for 16S rRNA amplification were adjusted as preliminary denaturation at 94°C for 5 min, then 40 cycles of 94, 56, and 72°C each for 30 s and a final step of extension at 72°C for 8 min. PCR products were then purified using QIAquick PCR Purification Kit (QIAGEN) and sequenced from MRC PPU DNA sequencing and services, University of Dundee, United Kingdom. Subsequently, obtained sequences were trimmed via BioEdit software and submitted to GenBank for getting accession numbers (Tables 1, 2).

**TABLE 1:** List of bacteria species produced straight chain monounsaturated fatty acids (^∗^*n*-MUFAs) as major group of cell membrane (CM) fatty acids (FA).

**Bacterial species**	**Accession No**	**% of *n*-MUFAs/CM FA**	**Major fatty acids (%/cell membrane FA)**	
*Deinococcus aquaticus* GW7	MK456560	78.3	*n*-C_15:1 (_*_*cis*_*_–10)_	32.9
*Massilia aurea* HI1	MK456563	84.3	*n*-C_15:1 (_*_*cis*_*_–10)_	37.1
*Massilia oculi* GI1	MK456529	58.5	*n*-C_16:1 (_*_*cis*_*_–9)_	50.8
*Pseudomonas brassicacearum* GS2	MK606638	69.4	*n*-C_16:1 (_*_*cis*_*_–9)_	35.8
*Pseudomonas migulae* GS3	MK606639	49.0	*n*-C_16:1 (_*_*cis*_*_–9)_	23.2
*Pseudomonas mandelii* GS12	MK606643	72.0	*n*-C_16:1 (_*_*cis*_*_–9)_	32.0
*Arthrobacter nitroguajacolicus* GS13	MK456545	89.6	*n*-C_16:1 (_*_*cis*_*_–9)_	42.0
*Paenisporosarcina macmurdoensis* GS17	MK456549	42.0	*n*-C_16:1 (_*_*cis*_*_–9)_	15.8
*Janthinobacterium lividum* GW1	MK456554	67.1	*n*-C_16:1 (_*_*cis*_*_–9)_	52.9
*Pseudomonas extremaustralis* HS8	MK456576	60.1	*n*-C_16:1 (_*_*cis*_*_–9)_	35.1
*Pseudomonas veronii* HS9	MK456577	72.2	*n*-C_16:1 (_*_*cis*_*_–9)_	25.8
*Pseudomonas fluorescens* HS10	MK456578	71.7	*n*-C_16:1 (_*_*cis*_*_–9)_	29.3
*Pseudarthrobacter sulfonivorans* HS14	MK456582	70.6	*n*-C_16:1 (_*_*cis*_*_–9)_	34.1
*Massilia timonae* HI7	MK456568	66.0	*n*-C_16:1 (_*_*cis*_*_–9)_	65.7
*Flavobacterium sinopsychrotolerans* GS7	MK456539	89.0	*n*-C_18:1 (*__tr__–9)_	50.8
*Paracoccus hibiscisoli* GS9	MK456541	94.4	*n*-C_18:1 (_*_*tr*_*_–9)_	93.2
*Brevundimonas vesicularis* GS11	MK456543	72.0	*n*-C_18:1 (_*_*tr*_*_–9)_	35.9
*Brevundimonas mediterranea* GS18	MK456550	77.2	*n*-C_18:1 (_*_*tr*_*_–9)_	58.6
*Brevundimonas intermedia* GS21	MK456553	83.7	*n*-C_18:1 (_*_*tr*_*_–9)_	57.5
*Sphingobium xenophagum* GhS4	MK456509	75.1	*n*-C_18:1 (_*_*tr*_*_–9)_	60.0
*Acinetobacter radioresistens* GhS8	MK456513	78.0	*n*-C_18:1 (_*_*tr*_*_–9)_	41.0
*Brevundimonas nasdae* GhW6	MK456525	77.5	*n*-C_18:1 (_*_*tr*_*_–9)_	58.2
*Sanguibacter antarcticus* GhW7	MK456526	53.6	*n*-C_18:1 (_*_*tr*_*_–9)_	45.5
*Rhizobium giardinii* HI5	MK456567	72.8	*n*-C_18:1 (_*_*tr*_*_–9)_	61.7

*Keys: ^∗^*tr*, trans; ^∗^*n*, straight chain.*

**TABLE 2:** List of bacteria species produced branched chain fatty acids (^∗^br-FAs) as major group of cell membrane (CM) fatty acids (FA).

**Bacterial species**	**Accession No**	**% of br-FA/CM FA**	**Major fatty acids (%/cell membrane FA)**	
*Enterobacter cloacae* GhS11	MK456516	73.4	^∗^*i*-C_15:0_	34.4
*Stenotrophomonas maltophilia* GhS14	MK456519	73.3	*i*-C_15:0_	32.3
*Arthrobacter agilis* GS1	MK456533	97.9	^∗^*a*-C_15:0_	58.2
*Rhizobium herbae* GS14	MK606644	96.6	*a*-C_15:0_	42.3
*Sporosarcina psychrophila* GS15	MK456547	82.2	*a*-C_15:0_	59.6
*Deinococcus depolymerans* GhS1	MK456506	50.6	*a*-C_15:0_	28.5
*Staphylococcus equorum* GhS5	MK456510	90.2	*a*-C_15:0_	55.4
*Arthrobacter sulfureus* GhS9	MK456514	98.5	*a*-C_15:0_	56.4
*Enterobacter mori* GhS12	MK456517	95.2	*a*-C_15:0_	58.9
*Plantibacter auratus* HI4	MK456566	89.0	*a*-C_15:0_	51.9
*Arthrobacter psychrolactophilus* HS1	MK456569	93.8	*a*-C_15:0_	60.3
*Pseudarthrobacter scleromae* HS2	MK456570	91.1	*a*-C_15:0_	47.7
*Bacillus butanolivorans* HS4	MK456572	84.0	*a*-C_15:0_	40.1
*Bacillus simplex* HS7	MK456575	93.8	*a*-C_15:0_	49.4
*Delftia acidovorans* HS13	MK456581	89.0	*a*-C_15:0_	59.3
*Pseudomonas frederiksbergensis* HW2	MK456589	88.4	*a*-C_15:0_	44.0
*Sphingomonas faeni* GW8	MK456561	96.6	*a*-C_17:0_	38.5
*Acidovorax radices* GW9	MK456562	98.3	*a*-C_17:0_	43.6

*Keys: ^∗^br, branched chain; ^∗^*i*, iso; ^∗^*a*, anteiso.*

### Production Media for Bacterial Growth

The experiments for the analysis of cell membrane fatty acids of all bacteria isolates were performed in Nutrient Agar (NA) [g L^–1^; D(+)-glucose 1, peptone 15, sodium chloride 6, yeast extract 3] and Reasoner’s 2A (R2A) (gL^–1^; casein acid hydrolysate 0.5, dextrose 0.5, dipotassium phosphate 0.3, magnesium sulfate 0.024, protease peptone 0.5, sodium pyruvate 0.3, starch soluble 0.5, yeast extract 0.5). The growth of isolates was conducted in 250 mL Erlenmeyer flasks with 50 mL production media at 15°C for 7 days. The biomass collection of bacteria isolates was achieved by centrifuging the cultures in 50 mL falcon tubes at 4,500 *g* at 10°C for 30 min. The resulting cell pellets were stored in 2 mL microcentrifuge tubes and freeze-dried prior to subsequent use.

### Preparation of Fatty Acid Methyl Esters (FAMEs)

Fatty acid methyl ester from all bacterial cell cultures were extracted using the protocol described by El Razak et al. (2014). Two mL of 5% methanolic HCl was mixed with 0.1 g of each dried bacterial cell culture and heated at 70°C in a water bath for 120 min in sealed glass tubes. The sealed glass tubes were allowed to cool at 23°C for 30 min. Then, 1 mL Milli-Q^®^ water was added to the tubes and vortexed, followed by 2 mL hexane and robust vortexing of the vial in order to extract the liberated fatty acid methyl esters (FAMEs). After phase separation, the solvent layer was transferred into a clean vial and the hexane removed under a gentle stream of nitrogen.

### Analysis of Cell Membrane Fatty Acid Distributions From Different Temperature Regimes

A total of 10 bacterial species, representing 10 different bacterial genera belonging to both Gram-negative and Gram-positive groups, were selected in order to determine the variation or similarity in composition of bacterial cell membrane fatty acids growing under different temperature regimes. The selection criteria of bacteria isolates was based on their ability to grow over a wide range of temperatures and their first time study on selected temperatures. Briefly, bacteria isolates were grown at 5, 15, 25, and 35°C using NB medium for 7 days. The bacterial biomass was obtained by centrifuging 40 mL falcon tubes containing bacterial cultures at 4,500 *g* at 10°C for 30 min. The pelleted bacterial cells were then subjected to FAME extraction as above.

### Gas Chromatography-Mass Spectrometry (GC-MS) Analysis

Fatty acid methyl esters derived from the bacteria isolates were analyzed by GC-MS. The GC-MS comprised a Thermo Scientific Trace 1300 gas chromatograph coupled via a heated transfer line to a Thermo Scientific ISQ LT single quadrupole mass spectrometer. The GC was fitted with a programmable temperature vaporising (PTV) injector and an Agilent-HP 1 capillary column coated with a 100% dimethylpolysiloxane stationary phase (50 m × 0.32 mm internal diameter × 0.17 mm film thickness). The carrier gas was helium at a flow rate of 2 mL min^–1^. The PTV was programmed to heat up to 300°C at 14°C sec^–1^, transferring the analytes onto the column. The GC temperature program started with an initial temperature of 50°C, held for 1 min, then heated to 100°C at 10°C min^–1^, then to 250°C at 4°C min^–1^ and finally to 300°C at 20°C min^–1^. The final temperature was maintained for 5 min. Compounds were identified by their mass spectra, retention times and elution order in comparison to a commercial reference standard (Sigma Aldrich C_4_-C_24_ FAME mix, inclusive of unsaturated compounds). Individual FAMEs were quantified by their peak areas relative to the peak area of an internal standard (methyl tetracosanoate, Sigma-Aldrich) and corrected for variable levels of ionization by a response factor that was calculated from analyses of the reference standard mix at the start of each analytical sequence. In addition, correlations between different temperature and fatty acids were also assessed in this study using GraphPad Prism 5.00.

## Results

In the current study, 42 different bacterial isolates representing 23 different genera were identified by 16S rRNA sequencing (Tables 1, 2). *Pseudomonas* was the most dominant genus followed by *Arthrobacter*, *Brevundimonas*, and *Massilia*. In addition, cell membrane fatty acids analyses revealed that in 24 out of 42 bacterial strains straight-chain monounsaturated fatty acids (MUFAs) were the major group of cell membrane (CM) fatty acid (FA), whereas 18 species were found to have branched fatty acids, both saturated and monounsaturated, as the dominant FAs in their cell membranes. The most important membrane fatty acids found in the 42 cold-tolerant bacterial species are listed in Tables 1, 2. The main individual *n*-MUFAs detected were *n*-C_15:1(_*_*cis*_*_–10)_, *n*-C_16:1(_*_*cis*_*_–9)_, and *n*-C_18:1(_*_*tr*_*_–9)_, whilst the dominant br-FAs are *i*-C_15:0_, *a*-C_15:0_, and *a*-C_17:0_. Most of the bacteria isolates were found to possess *a*-C_15:0_ (15 bacteria isolates) followed by *n*-C_16:1(_*_*cis*_*_–9)_ (12 bacteria isolates) and *n*-C_18:1(_*_*tr*_*_–9)_ (10 bacteria isolates) in their cell membranes. Only 1 species was found to produce *i*-C_15:0_, whereas 2 of each isolate produced *ai*-C_17:0_ and *n*-C_15:1(_*_*cis*_*_–10)_ as the primary cell membrane fatty acid moieties. Very few of the bacterial species studied produce polyunsaturated fatty acids (Table 3).

**TABLE 3:** Distribution of polyunsaturated fatty acids (PUFAs) in bacteria cell membranes at different temperatures.

**Bacterial species**	**PUFAs**	**μg g^–1^ (temperatures °C)**			
		**5**	**15**	**25**	**35**
*Flavobacterium sinopsychrotolerans* GS7	C_18:3(_*_*cis*_*_–6)_	0.28	5.41	ND^∗^	ND
	C_18:3(_*_*cis*_*_–9)_	0.15	0.27	ND	ND
	C_18:3 isomer_	0.11	ND	ND	ND
*Janthinobacterium lividum* GW1	C_17:3_	75.7	ND	ND	ND
	C_18:2(_*_*cis*_*_–9)_	1.99	ND	ND	ND
	C_18:2(_*_*tr*_*_–9)_	0.97	ND	ND	ND
	C_18:3(_*_*cis*_*_–6)_	0.22	ND	ND	ND
	C_18:3 isomer_	0.40	ND	ND	ND
*Brevundimonas nasdae* GhW6	C_18:2(_*_*cis*_*_–9)_	54.3	0.32	ND	ND
	C_18:2(_*_*tr*_*_–9)_	49.2	0.16	ND	ND
	C_20:3(_*_*cis*_*_–8)_	0.84	ND	ND	ND
	C_20:3(_*_*cis*_*_–11)_	1.05	ND	ND	ND
*Sphingomonas faeni* GW8	C_15:2(_*_*cis*_*_)_	21.7	ND	ND	ND
	C_15:2(_*_*tr*_*_)_	30.9	ND	ND	ND
	C_18:2(_*_*cis*_*_–9)_	0.09	0.14	ND	ND
	C_18:2(_*_*tr*_*_–9)_	0.04	0.34	ND	ND

*Keys: ^∗^not detected.*

In 32 out of 42 bacterial species, either br-FAs or *n*-MUFAs accounted for more than 70% of all quantified cell membrane FAs. In *Paracoccus hibiscisoli* GS9, *n*-MUFAs account for 94% of the determined FAs, followed by *Arthrobacter nitroguajacolicus* GS13 (89.6%), *Flavobacterium sinopsychrotolerans* GS7 (89.0%), *Massilia aurea* HI1 (84.3%) and *Brevundimonas intermedia* GS21 (83.7%), with 11 species showing relative amounts of *n*-MUFAs of more than 70%. The proportion of br-FAs reaches 98.5% in *Arthrobacter sulfureus* GhS9 followed by *Acidovorax radices* GW9 (98.3%), *Arthrobacter agilis* GS1 (97.9%), *Sphingomonas faeni* GW8 (96.7%), *Rhizobium herbae* GS14 (96.6%), *Enterobacter mori* GhS12 (95.2%), *Bacillus simplex* HS7 (93.8%), *Arthrobacter psychrolactophilus* HS1 (93.7%), *Pseudarthrobacter scleromae* HS2 (91.1%) and *Staphylococcus equorum* GhS5 (90.2%). The remaining 7 strains contain 70% br-FA in their cell membranes. Some strains show a dominance of a single compound such as *n*-C_18:1(_*_*tr*_*_–9)_ in *Paracoccus hibiscisoli* GS9 and *Rhizobium giardinii* HI5, with 93.3 and 61.7% of the quantified FAs, respectively.

In the current study, the effect of different temperatures on the distribution of fatty acids in the cell membranes of 10 bacterial species have been comprehensively determined (Table 4). The selected bacteria belong to Gram-negative (e.g., *Flavobacterium sinopsychrotolerans* GS7, *Paracoccus hibiscisoli* GS9, *Janthinobacterium lividum* GW1, *Sphingomonas faeni* GW8, *Brevundimonas nasdae*-GhW6, *Rhizobium giardinii* HI5 and *Pseudomonas extremaustralis*-HS8) and Gram-positive groups (e.g., *Sporosarcina psychrophila* GS15, *Staphylococcus equorum* GhS5 and *Arthrobacter psychrolactophilus* HS1). Correlations were used to represent association of temperatures with cell membrane fatty acids (Tables 5, 6). The Gram-negative strains accumulated straight-chain monounsaturated fatty acids (*n*-MUFAs) as the major group of cell membrane fatty acids except *Sphingomonas faeni* GW8 (Table 4). The highest quantity of *n*-MUFAs were produced at lower temperature, i.e., 5 and 15°C, and lower levels at higher temperature, i.e., 25 and 35°C, but *n*-MUFAs still formed the major group of cell membrane fatty acids at all temperatures, except for *Janthinobacterium lividum* GW1, which generated relative levels of straight-chain saturated fatty acids (*n*-SFA) of up to 58% at 35°C as compared to 36% of *n*-MUFAs. In addition, Gram-positive bacteria revealed predominantly branched-chain fatty acids, both saturated and monounsaturated, as the major groups among the analyzed cell membrane FAs (Table 4). However, at all temperatures, bacterial strains tend to incorporate higher levels of saturated branched-chain fatty acids into their cell membranes compared to branched-chain monounsaturated fatty acids (br-MUFAs). Saturated fatty acids occur only at very low levels (between 1.8 and 4%) in Gram-positive bacteria at all temperatures. In addition, Gram-positive bacteria exhibit the highest levels of saturated branched-chain fatty acids (br-SFAs) in their cell membranes at higher temperature, e.g., 25 and 35°C, and gradually decreasing levels of br-SFAs with decreased temperature, i.e., 5 and 15°C. Similarly, the highest levels of branched chain monounsaturated fatty acids were seen at low temperature (5 and 15°C) as compared to high temperature (25 and 35°C).

**TABLE 4:** Temperature derived distributions of the major groups of fatty acids in cell membranes of bacteria species.

**Bacteria**	**Temp (°C)**	**Percentage (%) of major groups of fatty acids**				
		***a. br*-SFAs**	***b. n*-SFAs**	***c. br*-MUFAs**	***d. n*-MUFAs**	***e.* PUFAs**
*1. Flavobacterium sinopsychrotolerans* GS7	5	0.4	10	0.6	89	–
	15	0.4	18.1	0.5	81	–
	25	0.2	35.6	0.2	64	–
	35	0.2	24.5	0.3	75	–
*2. Paracoccus hibiscisoli* GS9	5	4	5	1	89	1
	15	0.3	4	0.3	93.5	2
	25	0.1	6	0.1	93	–
	35	0.2	9.8	-	90	–
*3. Janthinobacterium lividum* GW1	5	–	27	0.2	68.8	4
	15	1.2	30	1.1	67	0.07
	25	5	40	–	55	–
	35	–	58	–	36	–
*4. Brevundimonas nasdae* GhW6	5	0.1	10	1.9	88	–
	15	0.3	20	1.7	78	–
	25	0.4	27	2.6	70	–
	35	0.5	33	2.5	64	–
*5. Rhizobium giardinii* HI5	5	–	1.9	10	88	–
	15	–	5.9	21	73	–
	25	0.1	15.9	20	64	–
	35	0.4	22.6	13	63	–
*6. Pseudomonas extremaustralis* HS8	5	26	8	7	59	–
	15	24	14	2	60	–
	25	11	30	1	58	–
	35	–	46.9	0.1	53	–
*7. Sphingomonas faeni* GW8	5	65	1	30	1	3
	15	80	1.9	16	2	0.1
	25	72	23	0.2	4.8	–
	35	7	51	–	42	–
*8. Sporosarcina psychrophile* GS15	5	85.3	1	13.2	1.5	–
	15	86.2	1	10.1	2.7	–
	25	90.6	2.4	3.4	3.6	–
	35	90	4	–	–	–
*9. Staphylococcus equorum* GhS5	5	71	2	19	8	–
	15	77	2	13	8	–
	25	86	5	7	2	–
	35	94	4	1	1	–
*10. Arthrobacter psychrolactophilus* HS1	5	85	0.4	14.5	0.1	–
	15	94	1.4	4.5	0.1	–
	25	98	1.5	0.5	–	–
	35	98	1.8	–	0.2	–

**TABLE 5:** Correlations of cell membrane fatty acids produced by Gram-negative bacteria species at different temperature.

**Correlations**					
**Fatty acids (mg/g)**	**Temperature (°C)**				**Correlation (*R^2^)***
	**5**	**15**	**25**	**35**	

***Rhizobium giardinii* HI5**					
*n-*C_16:0_	0.034	0.135	0.253	0.404	*0.991^∗∗^*
*n-*C_18:0_	0.085	0.163	0.252	0.369	*0.991^∗∗^*
*ai-*C_19:1_	0.315	0.606	0.803	0.961	*0.980^∗^*
*n-*C_16:1(_*_*cis*_*_–9)_	1.313	0.872	0.531	0.122	*0.997^∗∗^*
*n-*C_18:1(tr–9)_	1.116	1.248	1.501	1.694	*0.987^∗∗^*
***Brevundimonas nasdae* GhW6**					
*n-*C_15:0_	0.072	0.149	0.174	0.212	*0.943^∗^*
*n-*C_16:0_	0.195	0.423	0.520	0.724	*0.979^∗^*
*n-*C_17:1(tr–10)_	0.328	0.189	0.153	0.071	*0.944^∗^*
*n-*C_16:1(_*_*cis*_*_–9)_	0.643	0.371	0.114	0.070	*0.928*
*n-*C_18:1(tr–9)_	1.716	1.765	1.923	2.045	*0.964^∗^*
***Flavobacterium sinopsychrotolerans* GS7**					
*n-*C_16:0_	0.215	0.378	0.542	0.923	*0.948^∗^*
*n-*C_17:0_	0.036	0.046	0.150	0.233	*0.923*
*n-*C_16:1(_*_*cis*_*_–9)_	1.073	0.930	0.422	0.291	*0.936*
*n-*C_17:1(tr–10)_	0.757	0.536	0.145	0.115	*0.920*
*n-*C_18:1(tr–9)_	1.247	1.762	1.82	2.032	*0.874*
***Paracoccus hibiscisoli* GS9**					
*n-*C_16:0_	0.0003	0.001	0.004	0.010	*0.879*
*n-*C_18:0_	0.046	0.051	0.092	0.140	*0.911*
*ai-*C_15:0_	0.012	0.015	0.022	0.028	*0.977^∗^*
*i-*C_17:1_	1.200	0.946	0.03	0.014	*0.880*
*n-*C_18:1(tr–9)_	0.043	0.720	1.201	1.576	*0.982^∗∗^*
***Janthinobacterium lividum*** **GW1**					
*n-*C_16:0_	0.491	0.521	0.739	0.845	*0.932*
*n-*C_18:0_	0.003	0.005	0.04	0.059	*0.914*
*n-*C_16:1(_*_*cis*_*_–9)_	0.831	0.636	0.015	0.007	*0.884*
*n-*C_16:1(_*_*t*_*_*r–9)*_	0.015	0.058	0.268	0.404	*0.949^∗^*
*i-*C_17:0_	0.0006	0.001	0.023	0.017	*0.665*
***Sphingomonas faeni* GW8**					
*n-*C_16:0_	0.059	0.251	0.478	0.580	*0.979^∗^*
*n-*C_18:0_	0.015	0.401	0.540	0.613	*0.876*
*ai-*C_15:0_	0.573	0.279	0.124	0.063	*0.911*
*i-*C_16:0_	0.290	0.177	0.091	0.043	*0.969^∗^*
*ai-*C_17:0_	0.477	0.225	0.076	0.056	*0.880*
***Pseudomonas extremaustralis* HS8**					
*n-*C_16:0_	0.154	0.329	0.551	0.888	*0.977^∗^*
*i-*C_15:0_	0.228	0.161	0.129	0.004	*0.937*
*ai-*C_15:0_	0.432	0.231	0.094	0.0002	*0.972^∗^*
*ai-*C_17:1_	0.084	0.057	0.004	0.000	*0.918*
*n-*C_16:1(_*_*cis*_*_–9)_	0.961	0.751	0.676	0.618	*0.904*

*Keys: ^∗^Correlation is significant at the 0.05 level (two-tailed). ^∗∗^Correlation is significant at the 0.01 level (two-tailed).*

**TABLE 6:** Correlations of cell membrane fatty acids produced by Gram-positive bacteria species at different temperature.

**Correlations**					
**Fatty acids (mg/g)**	**Temperature (°C)**				**Correlation (*R^2^)***
	**5**	**15**	**25**	**35**	

***Staphylococcus equorum* GhS5**					
*n-*C_16:0_	0.005	0.009	0.034	0.062	*0.923*
*i-*C_15:0_	0.132	0.251	0.323	0.438	*0.991^∗∗^*
*ai-*C_15:0_	0.742	0.920	1.130	1.390	*0.992^∗∗^*
*i-*C_19:1_	0.165	0.094	0.032	0.009	*0.957^∗^*
*ai-*C_19:1_	0.255	0.177	0.061	0.002	*0.986^∗∗^*
***Sporosarcina psychrophila* GS15**					
*i-*C_15:0_	0.037	0.103	0.254	0.305	*0.962^∗^*
*ai-*C_15:0_	1.055	1.145	1.438	1.605	*0.964^∗^*
*ai-*C_17:0_	0.144	0.265	0.339	0.597	*0.933*
*i-*C_15:1_	0.342	0.279	0.102	0.0001	*0.970^∗^*
*ai-*C_15:1_	0.867	0.771	0.214	0.0004	*0.932*
***Arthrobacter psychrochitiniphilus* HS1**					
*n-*C_16:0_	0.002	0.011	0.02	0.041	*0.948^∗^*
*ai-*C_15:0_	0.805	0.994	1.101	1.186	*0.964^∗^*
*ai-*C_17:0_	0.635	0.635	0.794	0.874	*0.997^∗∗^*
*ai-*C_15:1_	0.350	0.165	0.013	0.004	*0.894*
*ai-*C_16:1_	0.014	0.008	0.004	0.0006	*0.982^∗^*

*Keys: ^∗^Correlation is significant at the 0.05 level (two-tailed). ^∗∗^Correlation is significant at the 0.01 level (two-tailed).*

Temperature greatly influences the distribution of individual fatty acids in cell membranes of all bacterial strains (Table 5). The straight-chain fatty acids such as C_15:0_, C_16:0_, C_17:0_, and C_18:0_ occur at higher levels at higher temperatures such as 25 and 35°C but at lower levels under lower temperatures, i.e., 5 and 15°C. Similarly, Gram-negative bacteria were observed to contain high levels of *n*-C_16:1(_*_*cis*_*_–9)_ and *n*-C_18:1(tr–9)_ with increasing temperature, although their production was also high at lower temperatures as well. Monounsaturated fatty acids such as *n*-C_16:1(_*_*tr*_*_–9)_ and *n*-C_17:1(_*_*tr*_*_–10)_ were also among those fatty acids whose production was enhanced at higher temperatures but decreased at low temperatures. Likewise, high levels of the branched saturated fatty acid, *i*-C_17:0_, were observed at 35°C but with lower levels at 5°C. *Sphingomonas faeni* GW8 produces *i*-C_16:0_ at higher levels at lower temperatures but at lower levels at 25 and 35°C. In addition, Gram positive bacterial strains were observed to generate the branched chain saturated component, *a*-C_15:0_, at high levels in their cell membranes. The levels of br-SFAs such as *i*-C_15:0_ and *a*-C_15:0_ gradually elevated with increasing temperature but with no sufficient difference. On the other hand, branched chain monounsaturated fatty acids such as *i*-C_15:1_, *a*-C_15:1_, *i*-C_19:1_ and *a*-C_19:1_ were produced by bacteria at lower levels at 25 and 35°C but found at higher levels at 5 and 15°C. Moreover, the straight chain fatty acids such as C_16:0_ and C_18:0_ were observed at high levels at increased temperature such as 25 and 35°C but in at low levels at lower temperature, i.e., at 5 and 15°C.

## Discussion

The present work aimed to study the distribution of saturated and unsaturated, normal and branched phospholipid fatty acids (PLFAs) in cell membranes of cold-tolerant bacterial strains belonging to both Gram-negative and Gram-positive groups. The comprehensive PLFA profiles of bacteria from Pakistan glaciers obtained in this study could be used to identify and quantify bacteria in cold habitats as each bacterial species displays a unique cell membrane fatty acid profile. Most importantly, the saturated C_16:0_ fatty acid is the most common FA, observed at high levels in all types of bacteria. Using PLFAs as bio-markers for bacterial identification and quantification continues to increase in popularity in the scientific community (Willers et al., 2015). PLFAs are the primary component of bacterial cell membranes; they degrade very rapidly upon cell death (White et al., 2009). Therefore, they are considered to be more representative of living bacterial biomass and more accurate than DNA-based approaches as PLFAs are more readily transformed following cell−death compared to DNA (Feinstein et al., 2009). Several researchers have used PLFA profiles for the quantification of bacteria in a given environment in order to understand function of genes involved in metabolic activities, for the screening of pathogenic bacteria, or the determination of community structures and bacterial diversity (Kellogg et al., 2001; Jungblut et al., 2009; Frostegard et al., 2011; Buhring et al., 2012; Naeher et al., 2012). Here we suggest that the comparison of PLFA distributions of bacteria isolated from cold environments with bacteria inhabiting warmer habitats, for identification purposes, would be misguided as fatty acid composition and quantity alter greatly alongside changes in the surrounding environment (Willers et al., 2015). However, PLFAs may be useful in association with the study of physiological changes in bacteria.

Most of the Gram-negative bacteria exhibit *n*-MUFAs as the major group of cell membrane FAs while those of Gram-positive species are dominated by br-SFAs, a characteristic pattern previously reported by Frostegard and Baath (1996). The *a*-C_15:0_, *n*-C_16:0_ and *n*-C_18:1_ as the dominant cell membrane FAs in Gram-positive species in the current study corroborates with the literature (Gillan et al., 1983; Breulmann et al., 2014; Dong et al., 2014; Fichtner et al., 2014; Reinsch et al., 2014). More specifically, *n*-C_16:1_ and the monounsaturated fatty acid *n*-C_18:1_ were the major types of fatty acid in cell membranes of the studied bacterial species summarized Table 1. Previous research has shown *n*-C_16:1_ and *n*-C_18:1_ as the most predominant PLFA and signature biomarkers of Gram-negative bacteria (Buckeridge et al., 2013; Djukic et al., 2013; Tavi et al., 2013; Zheng et al., 2013; Banks et al., 2014; Lange et al., 2014; Reinsch et al., 2014; Zhang et al., 2014).

Apart from general interspecies PLFA distributions we evaluated the effect of growth temperature on the relative distributions and levels of major FAs in cell membranes of Gram-negative and Gram-positive bacteria. Notably, increasing temperature caused a substantial decline in the relative amount of br-SFAs and br-MUFAs, in particular, in Gram-positive species (*Sporosarcina psychrophila* GS15, *Sphingomonas faeni* GW8, *Staphylococcus equorum* GhS5, *Arthrobacter psychrolactophilus* HS1) from about 90% at 5 and 15°C to less than 1% at 35°C while the amount of saturated normal FAs increased from an initial range of 0.4–2% to 1.8–51%. These observations corroborate the role of br-FAs in the adaptability of these bacteria toward changes in temperature. It has been reported that br-FAs maintain the normal liquid-crystalline (fluid) state of bacterial PLFA by lowering the melting point and, thus, enabling membranes to perform normal cellular function at low temperature (Suutari and Laakso, 1994; Bowles et al., 1996; Sun et al., 2012). An increased production of br-FAs by psychrophilic bacteria in response to lower temperatures has been observed previously, e.g., for the food pathogen *Listeria monocytogenes* (Annous et al., 1997). However, to the best of our knowledge, this is the first time that such temperature adaption is reported for bacteria species *Sporosarcina psychrophila*, *Sphingomonas faeni*, *Staphylococcus equorum*, and *Arthrobacter psychrolactophilus*. Other Gram-positive bacteria for which equivalent changes in PLFA composition at lower temperature has been observed include *Staphylococci*, incorporating increasing amounts of br-MUFAs into their cell membranes (Onyango and Alreshidi, 2018). Suutari and Laakso (1992) reported increased production of lower-melting point *a*-FAs at low temperature for *Bacillus subtilis* and *Bacillus megaterium* but decreased *i*-FA production. Similarly, Annous et al. (1997) reported an increase in levels of short-chain and *a-*branched-chain FAs in the cell membrane of *Listeria monocytogenes* with the temperature decreasing from 45 to 5°C.

Changes in FA distributions from different growth temperatures were similarly pronounced for the Gram-negative bacteria investigated, *Flavobacterium sinopsychrotolerans* GS7, *Paracoccus hibiscisoli* GS9, *Janthinobacterium lividum* GW1, *Brevundimonas nasdae* GhW6, *Rhizobium giardinii* HI5 and *Pseudomonas extremaustralis* HS8. In particular, the relative levels of saturated fatty acids such as C_15:0_, C_16:0_, C_17:0_, and C_18:0_ vary significantly. High levels of saturated FAs lower the membrane fluidity, thus, helping bacteria to survive at higher temperatures (Knothe and Dunn, 2009). Although *n*-MUFAs were the main group of membrane FAs of the investigated Gram-negative bacterial strains at all temperatures, the relative level of *n*-C_16:1(cis–9)_ decreased considerably with increasing temperature. The accumulation of MUFAs especially with *cis*-conformation has been reported as a means of lowering the FA melting point and improving cell membrane fluidity at low temperatures (Russell, 1989; Mangelsdorf et al., 2009). For example, saturated *n*-C_16:0_ has a melting point of 63°C whereas the introduction of one double bond, i.e., converting *n*-C_16:0_ to *n*-C_16:1_, lowers the melting point significantly to −1°C (Knothe and Dunn, 2009).

As far as we know, membrane FA adaption to growth temperature has not yet been reported for the above-mentioned Gram-negative species. Similar patterns were observed by Theberge et al. (1996) for the FA composition of *Rhizobium leguminosarum* at temperatures of 10, 15, 22, and 30°C, with higher levels of MUFAs and C_18:1_, in particular, at lower temperatures. Bajerski et al. (2017) evaluated the FA profile of *Chryseobacterium frigidisoli* and found monounsaturated *i*-C_17:1_ at high levels at lower temperature (10°C), confirming the role of unsaturated membrane FAs with a lower melting point in cold adaptation (Russell, 1984, 1989). In contrast, *Pseudomonas syringae*, which was isolated from ice samples of Antarctica, contains high levels of saturated and *trans*-monounsaturated FAs although fatty acids with the *cis*-isomers were also detected (Kiran et al., 2005).

Overall, the observed shifts in the distributions of saturated and unsaturated, normal and branched FAs with growth temperature appear consistent across the investigated 10 strains of Gram-positive and Gram-negative bacteria. This points toward the potential for the development of temperature-sensitive molecular ratios for (non-polar) glacial settings with high proportions of bacterial biomass such as glacial ice, melted water and sediments. Such approaches remain to be tested *in situ*.

## Conclusion

The analysis of cell membrane fatty acids of 42 cold-tolerant bacteria isolated from various glaciers of the Karakoram Mountain Range has revealed straight-chain monounsaturated fatty acids and branched-chain fatty acids as the dominant compounds among cell membrane fatty acids in Gram-positive and Gram-negative species, respectively. In addition, fatty acid distributions in bacterial cell membranes are significantly affected by growth temperature. In particular, a pronounced shift from enhanced proportions of monounsaturated to saturated branched and normal fatty acids with increasing temperature has been observed in Gram-negative bacteria species, whereas a notable increase in branched and normal saturated fatty acids with increased temperatures has witnessed in Gram-positive bacteria. Our study demonstrates the role of fatty acids for the maintenance of cell membrane fluidity in bacteria living in non-polar glacial habitats. The observed temperature-dependent shifts in fatty acid distributions may furthermore contribute to the development of temperature-sensitive molecular proxies in order to track temperature changes in (sub-) glacial environmental archives with high proportions of bacterial biomass. Finally, the capability of psychrophilic bacteria to produce biodesirable fatty acids, e.g., polyunsaturated fatty acids, represents an alternative source of such fatty acids for heterotrophic organisms in glacial environments.

## Data Availability Statement

All datasets generated for this study are included in the article.

## Author Contributions

NH, FH, and AA designed the research work. NH, MR, AH, and FH collected the samples from glaciers. AS, NH, and MR isolated and identified bacteria species. IB, JH, and NH carried out the GS/MS and afterward data analysis. FH and AA supervised the whole research work. FH and NH wrote the manuscript. All authors read and approved the final manuscript.

## Conflict of Interest

The authors declare that the research was conducted in the absence of any commercial or financial relationships that could be construed as a potential conflict of interest.
